# Spray-dried microencapsulation enhances the antioxidant efficacy of *Artemisia herba-alba* extract in butter: insights from volatile profiling and multivariate analysis

**DOI:** 10.1016/j.fochx.2026.104086

**Published:** 2026-06-08

**Authors:** Sara Lemmadi, Faiza Adoui, Emilie Descours, Pauline Chalut, Cyrille Santerre, Adem Gharsallaoui

**Affiliations:** a Laboratoire de Génie Agro-Alimentaire (GENIAAL), Institut de la Nutrition, de l'Alimentation et des Technologies Agro-Alimentaires (I.N.A.T.A.A.), Université Frères Mentouri Constantine 1 (UFMC1), Route de Ain El-Bey, Constantine 25000, Algeria; b Institut Supérieur International du Parfum, de la Cosmétique et de l'Aromatique Alimentaire (ISIPCA), 34-36 Rue du Parc de Clagny, 78000 Versailles, France; c Université Lyon 1, CNRS, LAGEPP, UMR 5007, 01000 Bourg-en-Bresse, France

**Keywords:** *Artemisia herba-alba*, Microencapsulation, Butter, Lipid oxidation, Volatile compounds, Natural antioxidants

## Abstract

The oxidative stability of butter enriched with an ethanolic extract of *Artemisia herba-alba* was evaluated in free and spray-dried microencapsulated forms. LC-HR-MS/MS identified 26 phenolic compounds, showing a total phenolic content of 94.55 ± 0.61 μg GAE/mg. Microcapsules were prepared using maltodextrin (DE19) and sodium caseinate. Four formulations (control, α-tocopherol 100 ppm, free extract 400 ppm, microencapsulated extract 400 ppm) were stored at 4 °C for 60 days. Oxidative stability was assessed through HS-SPME-GC–MS volatile profiling, CIELAB color analysis, sensory evaluation, and multivariate statistics. Lipid oxidation generated increasing levels of aldehydes, ketones, acids, and lactones, with the highest accumulation in control samples. While α-tocopherol showed the strongest protective effect, microencapsulation significantly reduced oxidation-related volatiles compared to the free extract and limited adverse sensory changes. These findings highlight spray-drying microencapsulation as an effective strategy to enhance the technological performance of plant phenolics in high-fat dairy systems.

## Highlights

• LC-HR-MS/MS identified 26 phenolic compounds in *Artemisia herba-alba* aerial part extract.
• Spray-drying microencapsulation improved antioxidant performance in butter.
• Encapsulated extract significantly reduced oxidation-related volatile compounds.
• Free extract negatively affected color and sensory attributes during storage.
• Multivariate analysis linked volatile markers with sensory and color changes.

## 1 Introduction

Butter is a high-fat dairy product widely consumed for its characteristic flavor and nutritional properties (Lee, 2020; Salehin et al., 2025). Structurally, butter is a water-in-oil (W/O) emulsion obtained by phase inversion of cream, in which proteins act as natural emulsifiers (Pouyamanesh et al., 2022). Its continuous lipid phase (approximately 80–82%) surrounds dispersed aqueous droplets (18–20%) (Cheng et al., 2023; Lee, 2020; Lokuge et al., 2024; Owusu-Apenten & Vieira, 2023). This specific multiphase organization plays a decisive role in its physicochemical stability. Butter contains mainly milk triacylglycerols composed of saturated and unsaturated fatty acids, together with cholesterol and fat-soluble vitamins (Mansour & Sindi, 2024). Despite its high proportion of saturated fatty acids (≈61%), the presence of unsaturated lipids (≈33%) renders butter highly susceptible to oxidative deterioration during storage (Demir et al., 2024; Mansour & Sindi, 2024).

Lipid oxidation is one of the primary reactions responsible for quality loss in butter (Asdagh & Pirsa, 2020; Demir et al., 2024; Pouyamanesh et al., 2022). In W/O systems, oxidation processes are often initiated at the water–oil interface, where oxygen, transition metals, and other pro-oxidant species may accumulate (Demir et al., 2024). The reaction between oxygen and unsaturated fatty acids leads to the formation of hydroperoxides, which subsequently decompose into secondary volatile compounds such as aldehydes, ketones, acids, lactones, and hydrocarbons (Clarke et al., 2020; Koyuncu & Tuncturk, 2017; Lokuge et al., 2024; Salehin et al., 2025). These molecules are directly associated with rancid off-flavors and sensory deterioration. Additionally, lipolytic bacteria may proliferate in the aqueous phase, producing lipases that hydrolyze triglycerides and further contribute to off-flavor development (Owusu-Apenten & Vieira, 2023). Oxidative degradation not only impairs organoleptic quality but also reduces nutritional value through the degradation of unsaturated fatty acids and fat-soluble vitamins, while potentially generating toxic oxidation products (Alwazeer et al., 2024; Asdagh & Pirsa, 2020; Lokuge et al., 2024; Mansour & Sindi, 2024).

To delay lipid oxidation, synthetic antioxidants such as butylated hydroxyanisole (BHA), butylated hydroxytoluene (BHT), tert-butylhydroquinone (TBHQ), and propyl gallate (PG) have traditionally been incorporated into fat-rich foods (Mohammadi et al., 2016). However, increasing regulatory scrutiny and consumer concerns regarding their potential cytotoxic, carcinogenic, and endocrine-disrupting effects have stimulated the search for safer alternatives (Ahmed et al., 2023; Mansour & Sindi, 2024). Consequently, plant-derived phenolic compounds have attracted considerable interest due to their radical-scavenging capacity and metal-chelating properties (Benamar-Aissa et al., 2023; Cheng et al., 2023).

Among medicinal plants rich in phenolic constituents, *Artemisia herba-alba* (commonly known as “Chih” in Algeria) has been widely investigated for its bioactive properties (Mrabti et al., 2023). Traditionally used to treat various disorders (Bourgou et al., 2017; Dhifallah et al., 2022; Sendi et al., 2020; Souhila et al., 2019), its aerial parts contain phenolic acids, flavonoids, tannins, sterols, essential oils, and sesquiterpene lactones, which contribute to its strong antioxidant potential (Almi et al., 2022; Bourgou et al., 2017). Although several studies have demonstrated the antioxidant activity of *A. herba-alba* extracts, their direct incorporation into food matrices remains limited.

Indeed, phenolic compounds are often sensitive to oxygen, light, heat, and pH variations, which may reduce their stability and efficacy during processing and storage (Hcini et al., 2021). Moreover, their direct addition can negatively affect sensory attributes. Microencapsulation, particularly by spray-drying, represents an effective technological approach to improve the stability, handling, and controlled release of bioactive compounds (Gómez-Gaete et al., 2024). Encapsulation can protect phenolic compounds against environmental degradation, enhance their dispersion within food systems (Leyva-Jiménez et al., 2020), and limit undesirable interactions with other food constituents (Delshadi et al., 2020). In addition, microencapsulation may increase the effective surface area of bioactive compounds (Chanioti et al., 2021; Leyva-Jiménez et al., 2020), promote their controlled release (Akbarmehr et al., 2023; Leyva-Jiménez et al., 2020), improve their antioxidant efficiency and stability, and reduce undesirable organoleptic impacts (Leyva-Jiménez et al., 2020). Although natural antioxidants have been explored in various lipid systems, the effectiveness of microencapsulated *A. herba-alba* extracts in a fat-continuous dairy matrix such as butter has not yet been reported. Considering the structural specificity of butter as a W/O emulsion and the critical role of oxidation-derived volatiles in quality deterioration, it is necessary to evaluate not only antioxidant efficiency but also its impact on sensory and color attributes.

Therefore, the objective of this study was to investigate the effects of incorporating α-tocopherol and *A. herba-alba* extracts, in both free and spray-dried microencapsulated forms, on the oxidative stability, volatile profile, color parameters, and sensory characteristics of sweet cream butter during 60 days of refrigerated storage at 4 °C.

## 2 Materials and methods

### 2.1 Materials and reagents

Sweet cream butter samples (82% butterfat) were purchased from the local supermarket (Carrefour brand, Massy, Cedex, France). The aerial parts of *A. herba-alba* were harvested from the Biskra region, Algeria. α-Tocopherol was obtained from Sigma-Aldrich (Steinheim, Germany). Maltodextrin (19 DE) was provided by Roquette-Freres SA (Lestrem, France), and sodium caseinate (92% protein) was supplied by Acros Organics (Geel, Belgium). Other chemicals were of analytical grade.

### 2.2 Ultrasound-assisted extraction of phenolic compounds from *A. herba-alba* aerial parts

For extracting phenolic compounds, ultrasound-assisted extraction (UAE) was used in accordance with the method proposed by our earlier research (Lemmadi et al., 2025). Briefly, 1 g of dried aerial parts of *A. herba-alba* powder was mixed with 20 mL of an ethanol/water solvent (80/20, *v/v*) in a conical tube. The obtained mixture was subjected to ultrasonic treatment in an ultrasonic bath sonicator (Jeken TUC-100, Dongguan, China) at 45 °C for 10 min, with a power of 240 W and a frequency of 40 kHz. The obtained ethanolic extract was centrifuged for 10 min at 5000 rpm, and then filtered through Whatman No. 1 filter paper. The solvent of extraction was then removed under reduced pressure using a rotary evaporator, set at 40 °C, followed by a laboratory freeze dryer at −54 °C for 24 h. The resulting dry extract was preserved at −18 °C until use.

### 2.3 Determination of total phenolic content (TPC)

TPC of *A. herba-alba* extract was evaluated using the Folin–Ciocalteu micro-method, as described by Reguig et al. (2025). In brief, 20 μL of extract solution was added to 100 μL of Folin-Ciocalteu reagent (previously diluted 1:10 with distilled water). A volume of 75 μL of sodium carbonate solution (7.5%, *w/v*) was added after 4 min. The mixture was then incubated at ambient temperature in the dark for 2 h, and absorbance was subsequently recorded at 765 nm with a 96-well microplate reader (Thermo Fisher Scientific, A51119600C, Illkirch-Graffenstaden, France). Gallic acid was utilized as a standard to establish the calibration curve. The TPC result was expressed as micrograms of gallic acid equivalents per milligram of dry extract (μg GAE/mg).

### 2.4 LC-HR-MS/MS analysis

Liquid chromatography-tandem mass spectrometry with high resolution (LC-HR-MS/MS) analysis of polyphenols in the ethanolic extract of *A. herba-alba* was performed using an 1260, Infinity II LC system (Agilent Technologies, France) coupled to a timsTOF mass spectrometer (Bruker, France) equipped with an electrospray ionization (ESI) source operated in negative ion mode. The chromatographic separation was carried out on a Poroshell 120SB C18 (2.1 × 150 mm 2.7 μm) column heated at 40 °C, the flow rate was set at 400 μL/min with water as solvent A and acetonitrile as solvent B with 0.1% of formic acid in A and B. Five μL of sample was injected using the following gradient: 0 min (5%B), 10 min (100%B), held for 4 min, 14.1 min (5%B) and 10 min of equilibration time. Ion source parameters were set as follows: capillary voltage 4.2 kV, nebulizer 3.0 bar, dry gas 10.0 L/min, desolvation temperature 250 °C. Nitrogen was used as drying gas in the source and for collision experiments. The instrument was daily calibrated in the 50–1350 *m*/*z* range using ESI-L low Concentration Tuning Mix solution (Agilent Technology). For Auto MS/MS experiments, parameters were set as follows: precursor ion range m/z 100–1320, No. of precursor 5, spectral rate 10.0 Hz, fixed CID 10 eV to 20 eV. The metabolites identified in this study were proposed by comparison with available MS/MS data and reported literature.

### 2.5 Microencapsulation of ethanolic extract by spray-drying method

Spray drying of *A. herba-alba* extract was performed according to the method suggested by our earlier studies (Lemmadi et al., 2025), using maltodextrin 19 DE (MD) and sodium caseinate as coating agents. 0.5% (*w/v*) of *A. herba-alba* extract was dissolved in distilled water and kept for 30 min under agitation at ambient temperature, protected from light. Then, 19% (*w/v*) maltodextrin 19 DE and 0.5% (*w/v*) of sodium caseinate were added to the active components solution. The mixture was stirred at ambient temperature in the dark for 30 min. The resulting mixture was dispersed using an Ultra-Turrax for 5 min at 10000 rpm, then was fed to a Mini Spray-Dryer (Buchi B-290, Switzerland) operating with the following experimental parameters: a flow rate of 500 mL/h, an air inlet temperature of 150 °C, and an air outlet temperature of 80 °C. The physicochemical and structural properties of the spray-dried microcapsules used in the present study were previously characterized in detail in our earlier work (Lemmadi et al., 2025). The microcapsules exhibited high encapsulation efficiency and strong antioxidant activity, and a predominantly spherical morphology, supporting their suitability for incorporation into fat-rich food matrices. The encapsulated extract obtained was stored at −18 °C in airtight amber vessels until further use.

### 2.6 Preparation of butter samples

Sweet cream butter samples (82% butterfat) were initially carefully mixed to eliminate batch effects and obtain a uniform bulk sample. The resulting bulk sample was then divided into four equal batches in order to prepare four distinct butter formulations, which were prepared as follows: butter enriched with 100 ppm of α-tocopherol (αTB), butter enriched with 400 ppm of unencapsulated extract of *A. herba-alba* (NEEB), butter enriched with 400 ppm of encapsulated extract of *A. herba-alba* (EEB), and butter without additives, which served as a control (CB). During preparation, each batch was homogenized using a blender at medium speed for 10 min. 250 g of each formulation was then repackaged in the same packaging as the butter initially purchased, then stored at 4 °C. The samples were stored for 60 days to perform oxidative stability tests compared with a control (CB), using various analytical methods, including determination of volatile compounds, color measurement, and sensory evaluation. Sampling was performed at 1, 30, and 60 days. Each volatile and colorimetric analysis was repeated three times for each sample.

### 2.7 Determination of volatile compounds

Volatile compounds were determined using headspace solid-phase microextraction (HS-SPME) coupled with chromatography-mass spectrometry (GC–MS). Each butter sample was analyzed in triplicate at each sampling point during refrigerated storage. Extraction of the volatile compounds was performed using HS-SPME analysis, according to a protocol optimized by Dadalı and Elmacı (2019), to capture the volatile organic compounds resulting from different types of oxidation during storage at 4 °C. Briefly, 2.5 g of each butter sample was weighed directly into 5 mL flat-bottomed glass vials sealed with Teflon-coated silicone caps, then placed on a heating block. SPME fiber (50/30 μm DVB/CAR/PDMS, Sigma-Aldrich, and Supelco, Darmstadt, Germany) was previously placed in the apparatus, then inserted into the vial and exposed to the headspace above the surface of the butter sample at a temperature of 48 °C for 34 min, and stirred at 300 rpm. After the extraction phase, a thermal desorption phase was performed using a GC-MS (7890B-5977B, Agilent Technologies Inc., USA), which aims to detect and separate the volatile organic compounds from butter. The thermal desorption of these volatile compounds was performed in the gas chromatography injector, where the saturated SPME fiber was removed from the vial and immediately inserted into the gas chromatography (GC) to desorb these volatile compounds. The desorption process lasted 2 min, and the GC injection conditions were set at 250 °C to ensure the total release of these volatile organic compounds. Helium gas (purity ≥99%) was chosen as the carrier gas. GC separation was performed on a DB-5MS capillary column (60 m × 0.25 mm i.d., 0.50 μm film thickness, Agilent Technologies, USA) in splitless mode with a carrier gas flow rate of 1.00 mL/min. The oven temperature was programmed as follows: heating to an initial temperature of 50 °C for 5 min, followed by a ramp with a flow rate of 8 °C/min to 240 °C and holding at 240 °C for 10 min. The MS source temperature was set to 250 °C, and the selective mass of the detector was operated in positive EI mode with a mass scan range of 50–550 *m*/*z* at an ionization energy of 70 eV.

Data were collected using GCMS Data Analysis software B.07.02.1938, examined and compared to the mass spectrometers libraries of ISIPCA, HPCH 2205, and NIST14 to identify each volatile component. The molecules identified by the mass spectrometer peaks were at least 80% similar to library data. The identified volatile compounds were semi-quantified using the Total Ion Chromatogram (TIC) peak area, which means as a percentage of the area of the total peak areas, allowing for comparison of each compound between samples.

### 2.8 Color measurement

Color of the butter samples was evaluated using a Minolta colorimeter (model CR-300, Konica Minolta Sensing Europe, Poissy Charles De Gaulle, France) equipped with D65 illuminant as a light source to determine CIELAB color parameter values, including the L* parameter values; which indicates the brightness of the sample in the range of (0) black to (100) white; a* and b* represent the greenness (negative values) to redness (positive values) and blueness (negative values) to yellowness (positive values) in the sample, respectively (Huang et al., 2024). Prior to collecting experimental measurements, the instrument was calibrated using black and white squares. The test was carried out in triplicate at ambient temperature for each sample at each sampling point (Clarke et al., 2020; Lee, 2020; Zhang et al., 2021). Results for each color parameter were expressed as the mean of three replicates ± standard deviation.

### 2.9 Sensory analysis

Sensory analysis of the minimally treated model food matrix of each butter formulation (CB, αTB, NEEB, and EEB) was carried out in the sensory laboratory of Institut Supérieur International du Parfum de la Cosmétique et de l'Aromatique alimentaire (ISIPCA). Sensory sessions were conducted in standardized sensory cabins at ambient temperature and under white lighting. The panel consisted of 10 staff panelists from ISIPCA, aged between 30 and 60 years. The analysis examined the change of butter's sensory attributes as affected by storage at 4 °C over intervals of 1, 30, and 60 days. Panelists conducted sensory evaluation through both a discriminative test (A-Not-A test) and a descriptive test. During each evaluation session, panelists were presented with 13 butter samples, each weighing 10 g, placed in transparent evaluation cups and coded with a random three-digit code.

#### 2.9.1 Discriminative test (A-Not-A test)

In this test, panelists assessed three industrial butter formulations enriched with different molecules (αTB, NEEB, and EEB) in comparison to the additive-free butter (CB). The objective was to assess whether the fortified samples could be distinguished from the control butter throughout the evaluated storage period (Marques et al., 2022).

#### 2.9.2 Descriptive test

In this descriptive test, each panelist received four butter samples in random order, accompanied by a slice of bread and a knife. The samples were evaluated based on three sensory attributes: color (yellow and green), smell (creamy, grassy, and rancid), and texture (spreadability). Evaluations were made on a 10-point scale, where 1 corresponded to « low intensity » and 10 to « high intensity » (Mallia et al., 2008).

All participants involved in the sensory evaluation were informed about the objectives and procedures of the study and provided their voluntary consent prior to participation. The sensory analysis was conducted anonymously, and all data were collected and processed in accordance with applicable privacy and confidentiality principles.

According to institutional guidelines for non-invasive sensory studies involving food products and voluntary adult participants, formal ethical committee approval was not required for the present study.

### 2.10 Data analysis

Statistical analysis of color and volatile compound data for the different butter formulations was conducted using a Tukey one-way ANOVA test with a 95% confidence interval using Minitab ® version 18 software. Chi-squared test (X^2^) was used to detect the difference between the A and Not-A samples for the data collected during the discriminative test conducted within the sensory analysis. In addition, a Multiple Factor Analysis (MFA) was performed to correlate the butter samples with the means of the assessed oxidation attributes (volatile compositions, color, and sensory characteristics) as a function of cold storage time using XLSTAT ® software (Sensory version 2021, Addinsoft, New York, NY, USA) where differences were defined as statistically significant at a level of *p* ≤ 0.05.

## 3 Results and discussions

### 3.1 Total phenolic content of *Artemisia herba-alba* aerial part extract

Phenolic compounds, a crucial group of plant secondary metabolites, have been recognized as effective natural antioxidants due to their ability to scavenge free radicals, chelate metals, and promote the expression of metabolic enzymes (Wang et al., 2023). The total phenolic content of the aerial part extract of *A. herba alba* was 94.55 ± 0.61 μg GAE/mg of dry extract. This result concurred with that obtained by Dhifallah et al. (2022) (98.92 ± 9.45 μg GAE/mg of dry extract). In our previous study (Lemmadi et al., 2025), both free and spray-dried encapsulated extracts exhibited strong *in vitro* antioxidant activity in DPPH assays, confirming the ability of the phenolic compounds to scavenge free radicals and supporting their potential application as natural antioxidants in lipid-rich food systems.

### 3.2 LC-HR-MS/MS

According to the LC-HR-MS/MS analysis, a total of 26 phenolic compounds were identified in the aerial part extract of *A. herba-alba* (Table 1), including 10 phenolic acids, 15 flavonoids, and one coumarin. Among the phenolic compounds identified, flavonoids, particularly flavones, were the predominant class with 12 compounds in the chemical profile of this extract. This result concurred with the findings of Bourgou et al. (2017). In addition, quinic acid, protocatechuic acid-4-*O*-β-glucoside, caffeic acid, and isoRhamnetin were identified for the first time in *A. herba-alba*.

**Table 1:** Phenolic profile identified by LC-HR-MS/MS in the non-encapsulated extract from the aerial parts of *Artemisia herba-alba*.

Class	RT (min)	Tentative identification	Molecular formula	[M–H]^−^ (m/z)	MS/MS fragmentation (m/z)	Ref.
Phenolic acids						
1	1.02	Quinic acid	C_7_H_12_O_6_	191.05	173.04, 127.04	(Carbonara et al., 2012; Zhang et al., 2016)
2	2.51	Protocatechuic acid-4-*O*-β-glucoside	C_13_H_16_O_9_	315.06	153.01	(Oh et al., 2023)
3	4.05	3-caffeoylquinic acid	C_16_H_18_O_9_	353.08	191.05	(Boudjelal et al., 2015)
4	4.09	5-caffeoylquinic acid)	C_16_H_18_O_9_	353.08	191.05	(Boudjelal et al., 2015)
5	4.30	4-caffeoylquinic acid)	C_16_H_18_O_9_	353.08	191.05	(Boudjelal et al., 2015)
6	9.74	Caffeic acid	C_9_H_8_O_4_	179.03	135.04	(Boukhalkhal et al., 2020; Carbonara et al., 2012; Li et al., 2017)
7	10.38	4-Feruloylquinic acid	C_17_H_20_O_9_	367.09	173.04	(Clifford et al., 2003)
8	10.44	3,4-di-caffeoylquinic acid	C_25_H_24_O_12_	515.10	353.08, 191. 05	(Boudjelal et al., 2015; Oh et al., 2023)
9	10.54	3,5-di-caffeoylquinic acid	C_25_H_24_O_12_	515.10	353.08, 191. 05	(Boudjelal et al., 2015; Oh et al., 2023)
10	10.73	3,4,5-Tricaffeoylquinic acid	C_34_H_30_O_15_	677.13	515.10, 353.07	(Younsi et al., 2016)

Flavonoids						
11	10.37	Vitexin (Apigenin-8-C-glucoside)	C_21_H_20_O_10_	431.08	341.05, 311.05	(Carbonara et al., 2012)
12	10.87	Luteolin (5,7,3′,4′-Tetrahydroxy flavone)	C_15_H_10_O_6_	285.03	267.02, 257.04, 243.02, 241.04, 217.04, 213.05, 199.03, 175.03, 151.00, 133.03	(Belguith-Hadriche et al., 2017)
13	10.88	5,3′-Dihydroxy-7,4′-dimethoxyflavanone	C_17_H_16_O_6_	315.04	300.02	(Souhila et al., 2019)
14	11.16	4′,5,7-Trihydroxy-3′,6- dimethoxyflavone	C_17_H_14_O_7_	329.06	271.02, 299.01, 314.03	(Fu et al., 2020; Oh et al., 2023)
15	11,17	Cirsiliol	C_17_H_14_O_7_	329,06	314.03, 299.01, 285.03, 271.02	(Souhila et al., 2019)
16	11.24	Irigenin	C_18_H_16_O_8_	359.06	344.04, 329.02, 314.00, 301.02	(Souhila et al., 2019)
17	11.31	Hispidulin (4′,5,7-Trihydroxy-6-methoxyflavone)	C_16_H_12_O_6_	299.05	284.03	(Xia et al., 2019)
18	11.35	Chrysoeriol (Lutoelin-3′-methyl ether or 5,7,4′-Trihydroxy-3′-methoxy flavone)	C_16_H_12_O_6_	299.05	284.03	(Boukhalkhal et al., 2020)
19	11.37	Jaceosidin (5,7,4′-trihydroxy-6′,5′-dimethoxyflavone)	C_17_H_14_O_7_	329.06	314.03	(Boukhalkhal et al., 2020; Okhlopkova et al., 2023)
20	11.39	Cirsimaritin (Cirsumaritin; Cirsitakaogenin; Scrophulein; 4′,5-Dihydroxy-6,7-Dimethoxyflavone; 6-Hydroxyapigenin-6,7-dimethyl ether)	C_17_H_14_O_6_	313.06	298.04, 283.02, 269.04, 255.02	(Souhila et al., 2019)
21	11.40	Diosmetin (3′,5,7-trihydroxy-4′-methoxyflavone)	C_16_H_12_O_6_	299.05	284.03	(Boukhalkhal et al., 2020)
22	11,51	Cirsilineol (4′,5-dihydroxy-3′,6,7-trimethoxyflavone, Fastigenin, Anisomelin, Eupatrin)	C_18_H_16_O_7_	343,07	313,03; 328,05	(Souhila et al., 2019)
23	11.56	Acacetin (apigenin-4′-methyl ether; 5,7-dihydroxy-4-methoxy flavone; Linarigenin; Buddleoflavonol)	C_16_H_12_O_5_	283.05	268.03	(Fu et al., 2020; Oh et al., 2023; Olennikov et al., 2019)
24	11.62	Genkwanin (Gengkwanin; Puddumetin; Apigenin 7-Methyl Ether)	C_16_H_12_O_5_	283.05	268.03	(Fu et al., 2020; Oh et al., 2023; Olennikov et al., 2019)
25	11.74	isoRhamnetin (Quercetin-3′-methylether; 3,5,7,4′-Tetrahydroxy-3′-methoxy flavone)	C_16_H_12_O_7_	315.04	300.02	(Boukhalkhal et al., 2020)

Coumarin						
26	10.92	Tomentin (5-hydroxy-6,7-dimethoxycoumarin or 6,7-dimethoxy-5-hydroxycoumarin)	C_17_H_14_O_8_	345.05	330.03, 315.00, 287.00, 259.02	(Souhila et al., 2019)

The same findings are reported in the study conducted by Souhila et al. (2019), who also identified, for the first time, vitexin, 5,3′-dihydroxy-7,4′-dimethoxyflavanone, tomentin, cirsiliol, irigenin, and chrysoeriol in the methanolic extract of the aerial parts of this medicinal plant, harvested in the Aïn Bel region (Algeria).

Complementing these findings, previous research performed by Dahmani-Hamzaoui et al. (2012) has shown that caffeoylquinic acid (CQA) derivatives identified in the extract of *A. herba-alba* growing wild in Algeria demonstrate their potent antioxidant properties, which could significantly contribute to this species being an interesting source of natural antioxidants that deserve to be used for potential food applications.

### 3.3 Volatile compounds

**Fig. 1:**
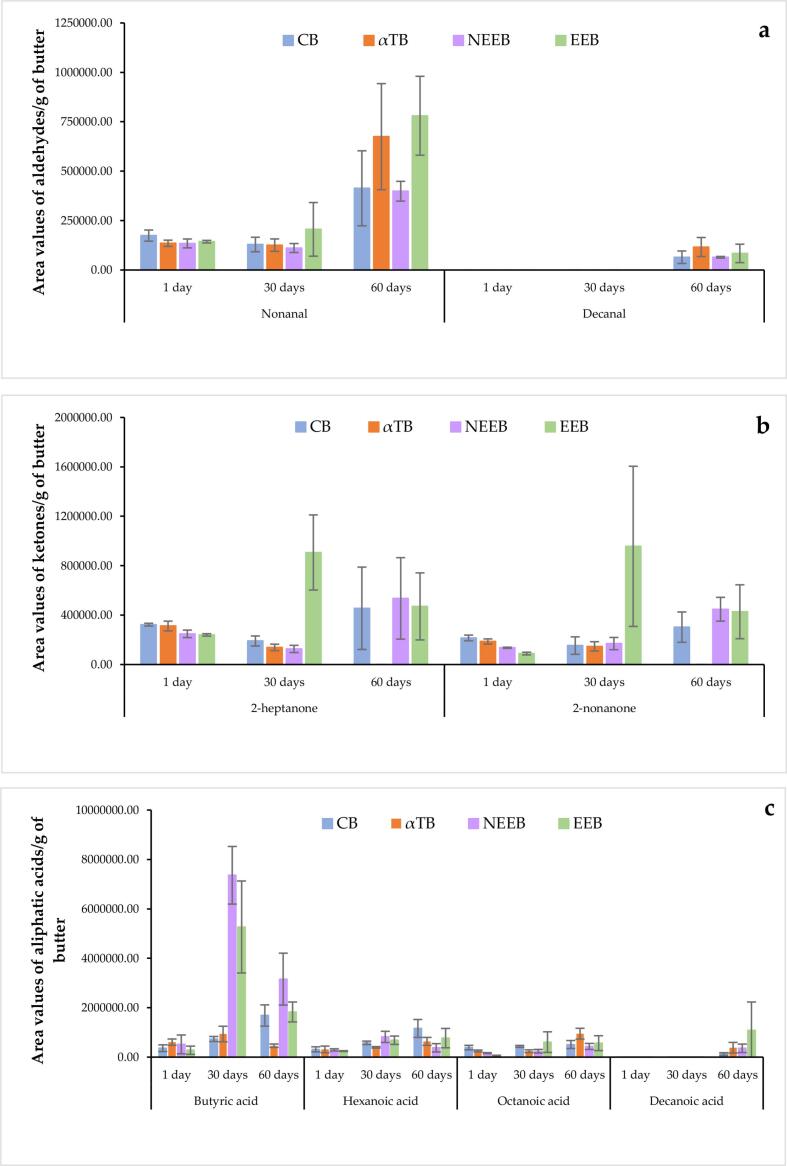

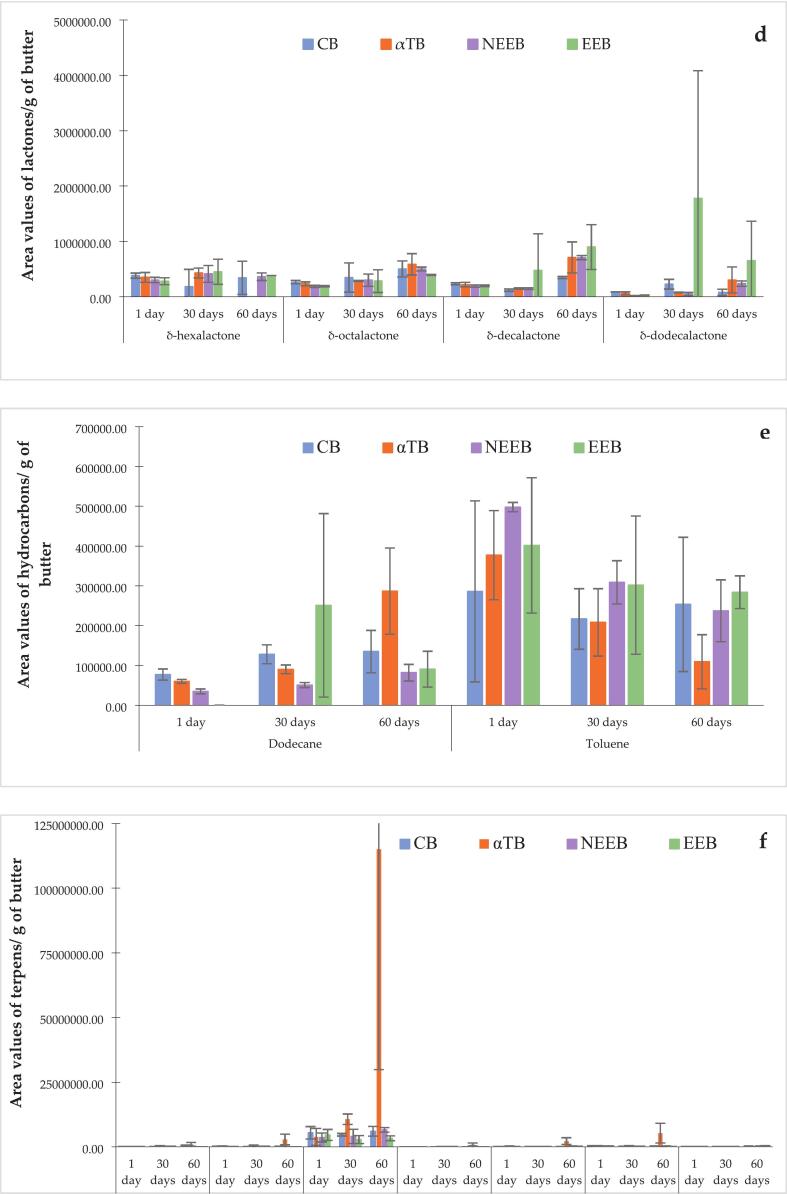
Evolution of volatile compounds area value detected per gram of butter samples evaluated as a function of refrigerated storage time. Data are represented as the mean ± standard deviation of three replicates.

Generally, lipid oxidation, microbial growth, and lipolysis levels are significant factors that influence butter quality during storage, leading to the generation of secondary oxidation products, mainly volatile carbonyl compounds, which contribute to the development of off-flavors (Alwazeer et al., 2024; Koyuncu & Tuncturk, 2017). Analysis of volatile compounds by the HS-SPME-GC–MS technique revealed a total of 21 volatile compounds in sweet cream butter with and without antioxidants, including 2 aldehydes, 2 ketones, 4 aliphatic acids, 4 lactones, 2 hydrocarbons, and 7 terpenes (Fig. 1).

Aldehydes are volatile compounds primarily associated with reducing food shelf life, as their production leads to deterioration of their organoleptic quality, especially their flavor (Koyuncu & Tuncturk, 2017). The aldehydes detected in butter samples include nonanal and decanal (Fig. 1a). Nonanal levels increased significantly in all butter samples during storage. This aldehyde is associated with the development of rancid flavor in lipid-rich foods (Grosso et al., 2018). However, as shown in Fig. 1a, decanal was only detected after 60 days of refrigerated storage. Similar results were obtained by Koyuncu and Tuncturk (2017), who reported that the concentrations of saturated aldehydes (pentanal, hexanal, and nonanal) increased in the butter samples analyzed over the evaluated storage times. Similarly, at the end of storage (after 60 days of refrigerated storage) (Fig. 1a), no significant difference was observed in nonanal and decanal area values among all butter samples evaluated (*p* > 0.05). In general, volatile aldehydes are produced by the cleavage of the carboxyl side of hydroperoxides, leading to the formation of an aldehyde and an acid (Demir et al., 2024). Furthermore, Lee (2020) reported that the oxidation of C18 unsaturated fatty acids, especially oleic acid (C18: 1n9), leads to the formation of aldehydes, including octanal, nonanal, and trans-2-decanal.

In addition, two ketones, 2-heptanone and 2-nonanone, were detected in all butter samples evaluated in this study (Fig. 1b). As shown in Fig. 1b, the area values of 2-heptanone showed no significant changes during storage time evaluated in the control (CB) and NEEB butter samples (*p* > 0.05), but showed fluctuating changes in EEB butter sample. Moreover, it showed a significant decrease in the αTB butter sample (*p* < 0.05). This finding may be explained by the antioxidant effect of α-tocopherol incorporated in the αTB butter formulation, which may retard oxidation reactions.

At the same time, no significant difference was observed in the area values levels of 2-nonanone in the EEB butter sample, which remained stable at each sampling point evaluated (after 1 day, after 30 days, and after 60 days of refrigerated storage) (Fig. 1b). On the other hand, its area value level increased significantly in the NEEB butter sample during the examined storage period (p < 0.05). This result can be attributed to the effectiveness of the encapsulation technique in protecting phenolic compounds from environmental factors and preventing their degradation, thus improving their stability in the incorporated food (Savan, 2023). Furthermore, it facilitates their dispersion in the food matrix (Leyva-Jiménez et al., 2020), which increases their contact surface and improves their bioactivity in butter compared to the unencapsulated extract. Likewise, the area values of 2-nonanone significantly decreased in the αTB butter sample throughout the refrigerated storage duration analyzed (*p* < 0.05). However, their area value remained stable in the control butter sample (CB) throughout the evaluated storage durations.

In this approach, Demir et al. (2024) indicated that ketones and aldehydes are the most common volatile compounds generated during lipid autoxidation. They can impart a fatty taste similar to paint when their concentration is high enough. They also indicated that even in small concentrations, ketones can provide typical aromas, such as floral and fruity. Furthermore, Erfani et al. (2020) indicate that the degradation of β-ketoacids leads to the formation of ketones through enzymatic activities or thermal processes. In contrast, Lee (2020) reported that straight-chain ketones mainly result from the oxidation of fatty acids or their thermal degradation.

Butyric, hexanoic, octanoic, and decanoic acids are volatile aliphatic acids detected in butter samples (Fig. 1c). Of which butyric and hexanoic acids were the predominant volatile acids detected in the evaluated butter samples (Fig. 1c). This result concurred with the reports of Erfani et al. (2020), Sarhir et al. (2021), and Demir et al. (2024), who declared that the main volatile acids present in butter were butanoic acid and hexanoic acid. Furthermore, based on the results shown in Fig. 1c, butyric acid, hexanoic acid, and octanoic acid were detected after one day of storage in all butter samples evaluated. On the other hand, decanoic acid was not detected in any butter samples before the 60th day of storage.

No significant difference was observed in the area values of butyric acid detected after one day of storage at 4 °C between all butter samples (CB, αTB, NEEB, and EEB) (*p* > 0.05). Likewise, a significant increase was observed in the area values of this acid in the NEEB and EEB butter samples up to 30 days of storage, followed by a significant decrease at the end of storage. On the other hand, the area values of this acid remained stable in the αTB sample throughout the evaluated storage period at 4 °C. However, a significant increase was observed in the area values of this evaluated acid in the control butter sample (CB) throughout the examined storage period (Fig. 1c). At the end of storage, the highest area value of butyric acid was detected in the enriched butter sample with unencapsulated extract of *A. herba-alba Asso* (NEEB), followed by CB and EEB samples, which had similar area values (p > 0.05), and finally by αTB sample, which had the lowest area value of butyric acid (Fig. 1c). This low area value detected in the αTB sample could explain the biological effect of α-tocopherol as a powerful antioxidant in the incorporated butter matrix.

Likewise, according to the results presented in Fig. 1c, after one day of refrigerated storage, no significant difference was detected in hexanoic acid area values between all butter samples analyzed (*p* > 0.05). Furthermore, after 60 days of storage at 4 °C, the butter sample enriched with the unencapsulated extract of *A. herba-alba Asso* (NEEB) showed the lowest hexanoic acid area value. This observation was attributed to the antioxidant properties of the phenolic compounds contained in this plant extract. On the other hand, the control butter sample (CB) showed a significant increase in hexanoic acid area values over storage time (p < 0.05), which contained the highest area value of this acid at the end of the examined refrigerated storage period compared with the other butter samples evaluated in this study (αTB, NEEB, and EEB).

Likewise, the octanoic acid data presented in Fig. 1c indicate that after one day of storage, the lowest area value of this acid was obtained in the butter sample enriched with the microcapsule powder containing the extract of *A. herba-alba Asso* (EEB). In contrast, the highest area value of this acid was observed in the control sample (CB). Furthermore, when monitoring the profile of volatile compounds resulting from butter oxidation, it was found that the area value of octanoic acid increased throughout the examined storage period for the αTB and NEEB samples. However, at the end of storage, no significant difference was observed in the area values of this acid between the different butter samples analyzed (CB, αTB, NEEB, and EEB) (*p* > 0.05).

Nevertheless, decanoic acid was detected only after 60 days of storage at 4 °C in all butter samples analyzed during this study (CB, αTB, NEEB, and EEB) (Fig. 1c), during which the four assessed butter samples showed similar area values of this acid (p > 0.05).

Previous research by Sarhir et al. (2021) demonstrated that the enzymatic activity of lipase of microbial lipolytic flora in butter induces the breakdown of triglycerides, leading to the generation of volatile aliphatic acids. Furthermore, Demir et al. (2024) discovered that the produced volatile aliphatic acids, particularly short–and medium-chain aliphatic acids, significantly affect the organoleptic quality of butter, particularly its flavor.

Lactones are aromatic compounds with an odor similar to that found in peaches and coconuts (Erfani et al., 2020). In this study, four lactones were detected in all butter samples after one day of refrigerated storage, including δ-hexalactone, δ-octalactone, δ-decalactone, and δ-dodecalactone. As can be seen in Fig. 1d, no significant difference was detected after one day of refrigerated storage in the area values of δ-hexalactone between all butter samples. Moreover, the area values of this lactone remained stable during the storage period for all studied butter samples, except for the αTB butter sample, where their area value decreased at the end of storage.

Among all samples, after one day of storage, the lowest area value of δ-octalactone was detected in the NEEB and EEB butter samples (Fig. 1d). Nevertheless, the highest area value of this lactone was found in the CB control butter sample. As shown in Fig. 1d, no significant variation in the area value levels of this lactone was observed in the EEB butter sample during storage time at 4 °C. This sample significantly retained the same area values of this lactone throughout the various storage periods examined (*p* > 0.05). In contrast, a significant increase was observed in δ-octalactone area values after 60 days of refrigerated storage in the αTB and NEEB butter samples. On the other hand, the control butter sample (CB) maintained similar area value levels for this lactone throughout all storage periods studied (p > 0.05).

However, after one day of refrigerated storage, there was no significant variation in the area values of δ-decalactone in the examined butter samples (CB, αTB, NEEB, and EEB) (p > 0.05), as shown in Fig. 1d. After 60 days of refrigerated storage, a significant increase was observed in the area values of δ-decalactone extracted from all the examined samples, except the EEB butter sample, which maintained similar area value levels of this lactone during all the examined sampling points (p > 0.05). This result can be attributed to the high distribution of encapsulated extract in the studied butter matrix, which enhances its powerful antioxidant effect, preventing oxidation reactions and delaying the accelerated formation of this lactone.

In this sense, the study of Lee (2020) revealed that the intensity of lactones (δ-octalactone, δ-decalactone, and δ-dodecalactone) increased in cow's cream butter with storage time. Furthermore, they found that the increase in the intensity of δ-decalactone and δ-octalactone in sweet cream butter with storage time could result from the hydrolysis of 4−/5-hydroxy fatty acids or the attack of oxygen on fatty acids at the 4- or 5-position, followed by hydrolysis. Furthermore, Erfani et al. (2020) indicated that lactones are generated by the hydrolysis of lactogenic glycerides to hydroxy acids through a thermal process. In contrast, Lee (2020) reported that γ/δ-lactones originate from thermal degradation or oxidation of fatty acids.

Hydrocarbons are by-products of lipid auto-oxidation and have an indirect impact on aroma, but they are involved in synthesizing other aromatic compounds (Demir et al., 2024). According to the results in Fig. 1e, two hydrocarbons, dodecane and toluene, were identified in the butter samples analyzed. After one day of storage at 4 °C, dodecane was detected in all butter samples, except the EEB butter sample, while toluene was detected in all butter samples analyzed (CB, αTB, NEEB, and EEB).

During the refrigerated storage period evaluated, the kinetics of dodecane formation in the control butter and butter enriched with encapsulated *A. herba-alba* extract (CB and EEB) samples remained significantly stable (Fig. 1e). In contrast, a significant increase was recorded in the kinetics of oxidation and the formation of this hydrocarbon in the αTB butter and NEEB butter samples during the refrigerated storage period studied (*p* < 0.05). At the end of the storage period studied, the lowest area value for this hydrocarbon was found simultaneously in the samples of butter enriched with unencapsulated extract and in those enriched with encapsulated extract (NEEB and EEB).

On the other hand, as shown in Fig. 1e, no significant difference in toluene area values was recorded after one day of refrigerated storage between all butter samples evaluated (CB, αTB, NEEB, and EEB) (p > 0.05). Furthermore, during the storage period analyzed, the levels of toluene area value in the butter sample enriched with encapsulated *A. herba-alba* extract and the control butter (EEB and CB) remained stable throughout the refrigerated storage period evaluated. In contrast, a significant decrease was observed in the levels of the area values of this hydrocarbon detected during the refrigerated storage times studied in the NEEB and αTB butter samples (*p* < 0.05). At the end of the storage period, no significant difference was observed between the area values of this hydrocarbon formed in all butter samples analyzed.

Erfani et al. (2020) demonstrated that the oxidation of saturated fatty acids leads to the formation of hydrocarbons. On the other hand, Demir et al. (2024) reported that hydrocarbon generation results from the cleavage of the methyl side of hydroperoxides, leading to the formation of a hydrocarbon and an oxoacid.

α-pinene, β-pinene, d-limonene, sabinene, β-myrcene, P-cymene, and γ-terpinene are terpenes identified in butter samples. Among the seven terpenes detected in the butter samples, d-limonene was the predominant (Fig. 1f). Terpenes are secondary metabolites derived from plants included in forage mixtures intended for grazing animals (Sulejmani et al., 2014). Of which, their quantities in milk and dairy products can vary depending on different factors such as geographical region, season, and the variety of plants contained in the animal's diet (Demir et al., 2024). As can be seen in Fig. 1f, no significant differences were observed in the area values of all terpenes identified in each butter sample as a function of refrigerated storage time, except for the αTB butter sample, where the area values of α-pinene decreased with refrigerated storage time. Meanwhile, the area values of sabinene and β-myrcene increased over time. In contrast, their γ-terpinene area values showed fluctuating variations during storage. At the end of storage, the area value of γ-terpinene found in the αTB butter sample was generally significantly lower than its initial value.

### 3.4 Color analysis

Table 2 presents the changes in color parameter values of butter samples during the refrigerated storage period examined at 4 °C. According to Commission Internationale de l'Éclairage, the L* parameter values represent the brightness ranging from black (0) to white (100); a* and b* represent the change in color from green (negative values) to red (positive values) and from blue (negative values) to yellow (positive values) in the sample, respectively (Lee, 2020). Based on the L* parameter values shown in Table 2, after one day of storage, the CB and αTB butter samples were whiter (higher CIE L* values) than the NEEB and EEB butter samples (p < 0.05). In addition, a slight brightening was observed after 30 days of storage at 4 °C, reflecting a slight increase in the values of this color parameter (CIE L*) for all the butter samples examined in this study (CB, αTB, NEEB, and EEB). Subsequently, a significant decrease was observed in these parameter values after 60 days of storage for CB, αTB, NEEB, and EEB.

**Table 2:** Evolution of color of butter samples during refrigerated storage.A-D, a-c

Color parameter	Sample	Storage (days)		
		1	30	60
L*	CB	84.94 ± 0.20^Ab^	86.20 ± 0.03^Ba^	81.63 ± 0.01^Cc^
	αTB	85.80 ± 0.28^Ab^	87.30 ± 0.01^Aa^	83.74 ± 0.06^Ac^
	NEEB	83.65 ± 0.39^Bb^	84.46 ± 0.01^Ca^	82.67 ± 0.02^Bc^
	EEB	82.45 ± 0.52^Cb^	83.28 ± 0.01^Da^	81.07 ± 0.02^Dc^
a*	CB	−0.83 ± 0.07^Ac^	0.66 ± 0.23^Ab^	1.03 ± 0.00^Aa^
	αTB	−1.17 ± 0.09^Bc^	0.05 ± 0.01^Bb^	0.44 ± 0.01^Ba^
	NEEB	−1.31 ± 0.01^Bc^	−0.25 ± 0.00^Cb^	−0.02 ± 0.01^Da^
	EEB	−1.19 ± 0.16^Bc^	0.43 ± 0.01^Aa^	0.05 ± 0.01^Cb^
b*	CB	21.40 ± 0.58^Aa^	22.17 ± 0.12^Ca^	22.25 ± 0.01^Ca^
	αTB	18.81 ± 0.15^Bc^	20.52 ± 0.02^Db^	22.76 ± 0.01^Ba^
	NEEB	21.02 ± 0.13^Ac^	23.16 ± 0.01^Ba^	22.06 ± 0.03^Db^
	EEB	22.00 ± 0.77^Ab^	24.19 ± 0.01^Aa^	24.28 ± 0.01^Aa^

A-D : Different capital letters indicate a significant difference between the values of the same color parameter for different butter samples in the same column for the same storage days (p < 0.05).

a-c : Different lowercase letters represent a significant difference between the values of the same color parameter for the same butter samples in the same rows for different storage times (p < 0.05).

As shown in Table 2, the a* parameter values demonstrate that after one day of storage, the αTB, NEEB, and EEB butter samples recorded the highest green color intensities compared to the control butter (CB), which were − 1.17, −1.31, −1.19, and − 0.83, respectively. In addition, a progressive increase in the values of this parameter was observed during the storage period for all analyzed butter samples (CB, αTB, NEEB, and EEB). At the end of storage and after 60 days of refrigerated storage, the lowest value for this parameter, a*, was recorded for the NEEB butter sample (−0.02), followed by EEB (0.05), then αTB (0.44), and finally CB (1.03). This finding demonstrates that the unencapsulated extract has a significant impact on the organoleptic quality of the butter matrix, particularly in terms of color, resulting in a greener butter color compared to the encapsulated extract.

As can be seen in Table 2, the b* parameter data for the different butter samples were positive values that fell within the yellow color range. After one day of storage, the lowest value for this color parameter (b*) was recorded for the αTB butter sample (18.81). In addition, a significant increase in yellow color intensity was observed throughout the evaluated refrigerated storage period for all butter samples examined, except for the control butter sample (CB), which remained stable during the 60-day storage period. The results of this study indicate that incorporating α-tocopherol, unencapsulated extract, and encapsulated extracts into butter could lead to higher b* values than in the control samples. These results for the b* parameter are similar to those obtained by Alwazeer et al. (2024), who reported that the b* values of different butter formulations enriched with H_2_, N_2_, and BHT gradually increased during storage time. This result could be attributed to the protection of liposoluble carotenoids from oxidation reactions. Indeed, the electron-rich conjugated double bond structure of β-carotene makes it unstable to oxidation (Alwazeer et al., 2024). Furthermore, at the end of storage, the highest value for this b* parameter was recorded for the butter sample enriched with encapsulated extract of *A. herba-alba*. This finding may be attributed to the spray-drying microencapsulation technique, which enables the production of encapsulated extracts while preserving their bioactivity and stability in the evaluated food matrix, thereby preventing carotenoid oxidation. These results improved the preservation of the yellow color of butter compared to the unencapsulated extract.

In this approach, the study conducted by Zhang et al. (2021) reported that the variation in the overall color of palm oil samples could be attributed mainly to the conversion of trans carotenoids to cis isomers, the generation of oxidation products, and the production of volatile degradation products and other low molecular weight compounds. Furthermore, the study performed by Alwazeer et al. (2024) indicated that oxidation reactions of pigments and fats are the leading cause of color change in foods. These findings are why the use of antioxidants is a common practice to prevent oxidation reactions in food products.

### 3.5 Sensory evaluation

#### 3.5.1 Discriminative test

Table 3 shows the Chi-squared values calculated for each sensory evaluation session, where the panelists performed this discriminative test. Based on the data presented in Table 3, the null hypothesis was rejected in the first and the third sensory evaluation sessions (after one days' storage and after 60 days storage at 4 °C), where the Chi-squared values calculated were 10.26 and 5.88, respectively, which are more significant than the critical Chi-squared value with a 5% risk of the two-sided hypothesis at the first degree of freedom (which was 3.84), reflecting the fact that a significant difference was detected between the control butter sample (A = CB) and the other butter formulations (Not-A = αTB, NEEB, and EEB) by panelists.

**Table 3:** Chi-squared values calculated for each sensory evaluation session.

	Storage time (days)		
	1	30	60
Chi-squared value (x^2^)	10.26	0.93	5.88

In contrast, the data in Table 3 show that the Chi-squared value calculated in the second sensory evaluation session (after 30 days' storage) was 0.93, which was inferior to the critical Chi-squared value at 5% risk. This result revealed that no significant difference was detected between the A and Not-A samples, meaning that panelists perceived no difference between the butter samples without additives and those enriched with α-tocopherol, ENE, and EE, after 30 days of storage.

#### 3.5.2 Descriptive test

**Fig. 2:**
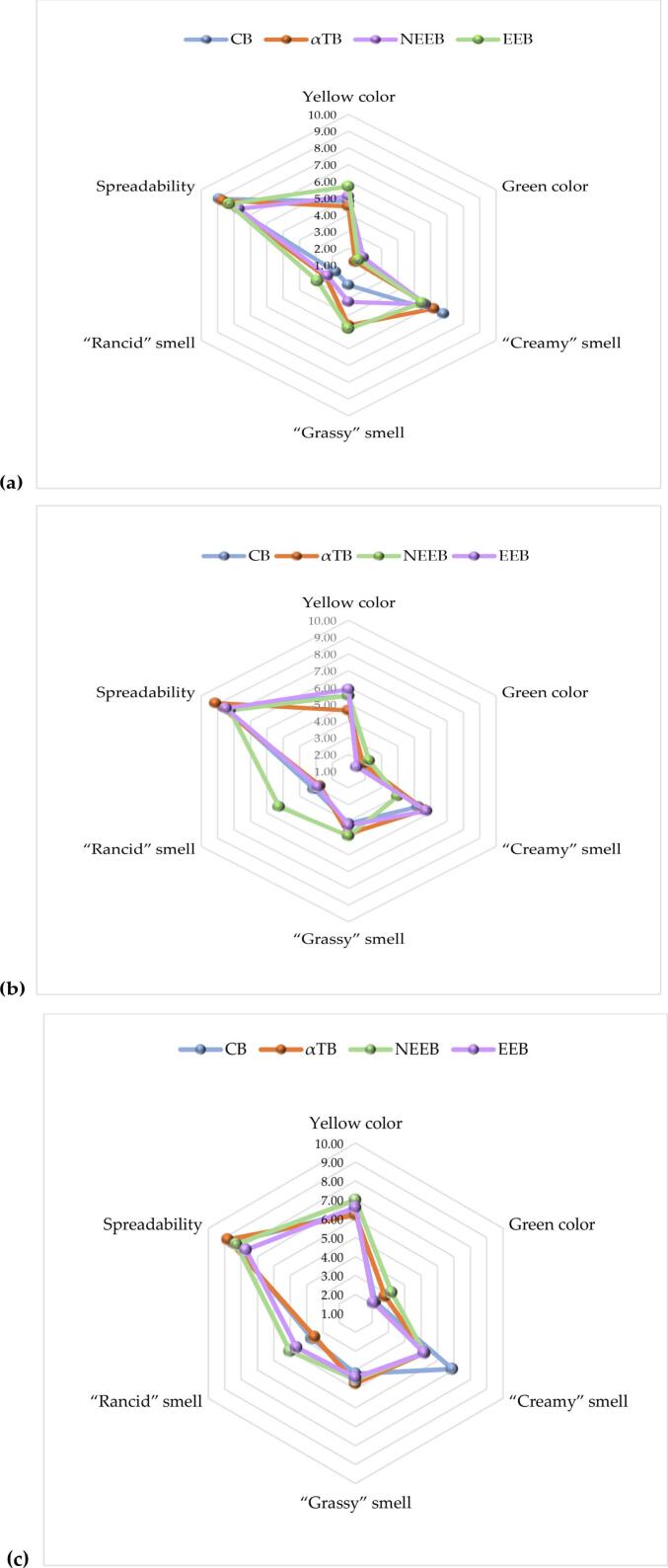
Evolution of the sensory profile of different butter samples during refrigerated storage, evaluated by descriptive test: (a) after one day of storage, (b) after 30 days of storage, and (c) after 60 days of storage.

Fig. 2a-c shows the sensory characteristics of the butter samples examined as a function of refrigerated storage times. During the first day of storage, all butter samples received almost identical average scores for all attributes evaluated, except the attribute “grassy smell“, which was more pronounced for the αTB, NEEB, and EEB butter samples than for the control butter sample (CB). This finding could be due to the antioxidant compounds (α-tocopherol and unencapsulated and encapsulated extract of *A. herba-alba*) incorporated into these butter formulations.

However, after 30 days of storage, the average score given by panelists to the “creamy odor” attribute of the butter sample enriched with unencapsulated *A. herba-alba* extract (NEEB) was lower than that given to the CB, αTB, and EEB butter samples. In addition, the average score given by panelists for the “rancid odor” attribute for the same NEEB sample was higher than that given to the CB, αTB, and EEB samples (Fig. 2b). These results clearly demonstrated the negative impact of directly incorporating unencapsulated plant extract on the sensory quality of the food product studied, particularly its odor. Direct incorporation limits the bioactivity of this plant extract, reflecting low antioxidant activity against oxidation reactions in the studied food matrix, which affects the overall stability of the butter. Consequently, butter enriched with unencapsulated extract had a more pronounced rancid odor than butter enriched with encapsulated extract. The increase in rancid odor scores observed in the NEEB butter sample compared to the EEB sample is consistent with the results for volatile compounds obtained by HS-SPME-GC–MS analysis.

Furthermore, as shown in Fig. 2c, after 60 days of storage, the panelists gave slightly higher average scores for the “rancid odor” attribute to the NEEB and EEB samples than to the αTB and CB samples.

### 3.6 Multiple factor analysis (MFA)

**Fig. 3:**
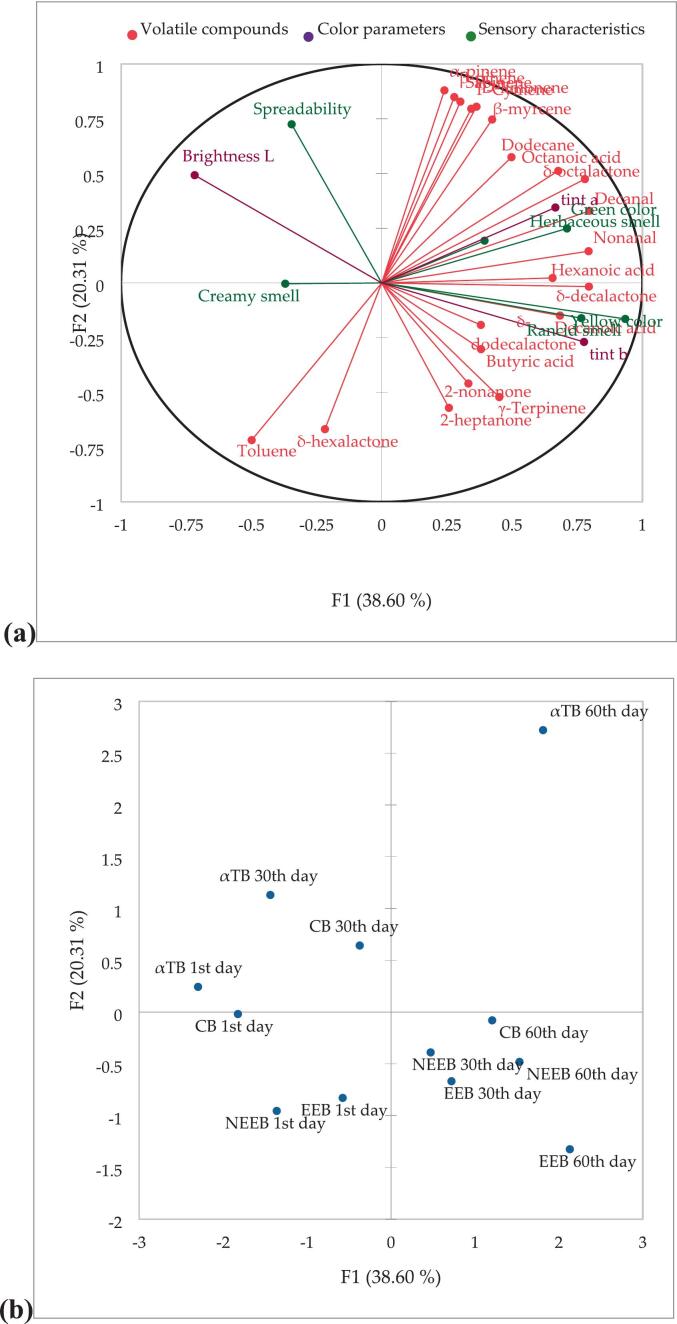

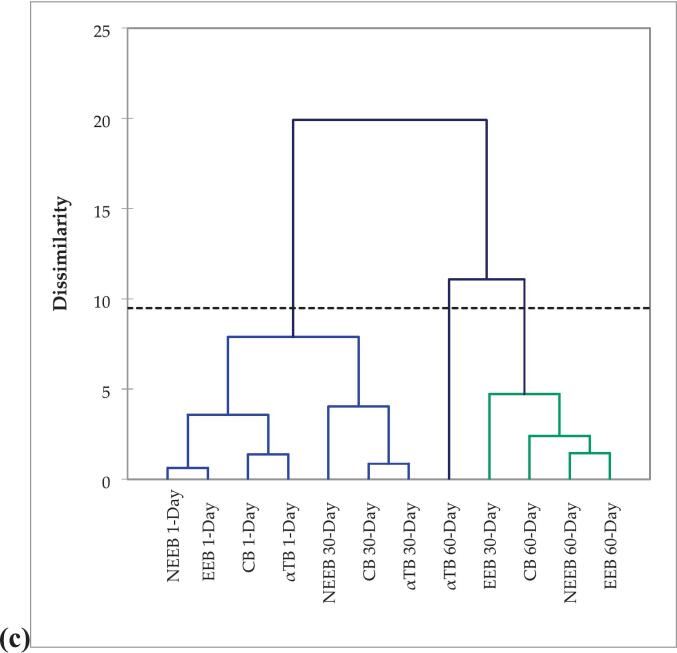
Multiple factor analysis of oxidative, color, and sensory parameters of different butter samples during refrigerated storage.

MFA was applied to the data of the different groups to distinguish the effects of the control, treatment (αTB, NEEB, and EEB), and storage times (1 day, 30 days, and 60 days) on the volatile compounds, color, and sensory characteristics of all butter samples. This approach aimed to highlight potential correlations among parameters measured during HS-SPME-GC–MS, color, and sensory analyses, as well as to establish the relationships between these parameters and changes in the preservation profiles of the different butter samples over the storage period (Fig. 3a).

Two principal axes, F1 (38.60%) and F2 (20.31%), were extracted by MFA, explaining together 58.91% of the total variance in volatile compounds, color parameters, and sensory characteristics across all butter samples. Axis F1 mainly separated sensory descriptors (in red) from volatile compounds (in green) and color parameters (in purple), suggesting that certain molecular classes are strongly associated with particular sensory perceptions. This organization also highlights a close correlation between color parameters and certain specific sensory perceptions. Axis F2 represents secondary variations that can be attributed to structural differences between volatile compounds and their impact on sensory perceptions, as well as linked variations between color parameters and their sensory impact. Fig. 3a shows that the sensory descriptors “rancid odor” and “yellow color” are closely associated with molecules such as decanoic acid, δ-decalactone, and δ-dodecalactone, as well as the color parameter “tint b.” This observation suggests that these volatile compounds and this color parameter may contribute significantly to these sensory perceptions. Furthermore, the sensory descriptors “grassy odor” and “green color” are strongly linked to terpene molecules and the color parameter “tint a”. This finding suggests that these volatile compounds, along with this color parameter, may contribute to these sensory perceptions. Conversely, the sensory descriptors “creamy smell” and “Spreadability” are negatively correlated with certain chemical compounds, such as toluene and δ-hexalactone, indicating a low contribution from these molecules.

In Fig. 3b and c, the distribution of butter samples according to the variables analyzed during storage was examined. In these plots, samples treated with NEE and EE on day 60 were placed in the same subgroup. In contrast, the butter sample treated with α-tocopherol clearly stands out from the other treatments.

## 4 Conclusion

In this study, the phenolic profile of *Artemisia herba-alba* aerial part extract was characterized and its antioxidant performance was evaluated in sweet cream butter, either in free form or after spray-drying microencapsulation. LC-HR-MS/MS analysis identified 26 phenolic compounds, predominantly flavonoids and caffeoylquinic acid derivatives, supporting the strong antioxidant potential of the extract. During 60 days of refrigerated storage, volatile profiling revealed progressive lipid oxidation in all samples, with the highest accumulation of oxidation-related aldehydes, ketones, acids, and lactones observed in the control butter. While α-tocopherol exhibited the strongest protective effect, microencapsulated extract significantly reduced the formation of key oxidation markers compared to the free extract. In contrast, direct incorporation of the unencapsulated extract provided limited oxidative protection and negatively affected color parameters and sensory attributes, particularly green tint and grassy/rancid notes.

Microencapsulation improved the technological performance of *A. herba-alba* phenolics within the fat-continuous butter matrix, likely by enhancing their stability and dispersion while limiting undesirable sensory impacts. Multivariate analysis confirmed the strong correlation between oxidation-derived volatile compounds and changes in color and sensory properties.

Overall, spray-drying microencapsulation represents a promising strategy to enhance the applicability of plant-derived phenolic antioxidants in high-fat dairy systems. Nevertheless, the present study has certain limitations, particularly the absence of a blank microcapsule control and complementary classical lipid oxidation indices, such as peroxide and p-anisidine values, which could provide a more comprehensive understanding of oxidation mechanisms and the respective contributions of wall materials and encapsulated phenolic compounds. Future studies should also investigate release behavior and long-term oxidative stability under different storage conditions. Despite these limitations, the present results support the potential of encapsulated *A. herba-alba* extract as a natural alternative for improving oxidative stability and maintaining butter quality during storage.

## CRediT authorship contribution statement

**Sara Lemmadi:** Writing – original draft, Investigation, Formal analysis, Data curation. **Faiza Adoui:** Writing – review & editing, Validation, Supervision, Project administration, Funding acquisition. **Emilie Descours:** Writing – review & editing, Investigation, Formal analysis. **Pauline Chalut:** Writing – review & editing, Investigation, Formal analysis. **Cyrille Santerre:** Writing – review & editing, Investigation, Formal analysis. **Adem Gharsallaoui:** Writing – review & editing, Validation, Supervision, Project administration, Funding acquisition.

## Declaration of competing interest

The authors declare that they have no known competing financial interests or personal relationships that could have appeared to influence the work reported in this paper.

## Data Availability

Data will be made available on request.
